# Atrophy of the optic chiasm is associated with microvascular diabetic complications in type 1 diabetes

**DOI:** 10.3389/fendo.2023.1134530

**Published:** 2023-05-30

**Authors:** Aleksi Tarkkonen, Tor-Björn Claesson, Marika I. Eriksson, Carol Forsblom, Lena M. Thorn, Paula Summanen, Per-Henrik Groop, Jukka Putaala, Daniel Gordin, Juha Martola

**Affiliations:** ^1^ HUS Medical Imaging Center, Radiology, University of Helsinki and Helsinki University Hospital, Helsinki, Finland; ^2^ Folkhälsan Institute of Genetics, Folkhälsan Research Center, Helsinki, Finland; ^3^ Department of Nephrology, University of Helsinki and Helsinki University Hospital, Helsinki, Finland; ^4^ Research Program for Clinical and Molecular Metabolism, Faculty of Medicine, University of Helsinki, Helsinki, Finland; ^5^ Department of General Practice and Primary Health Care, University of Helsinki and Helsinki University Hospital, Helsinki, Finland; ^6^ Department of Ophthalmology, University of Helsinki, Helsinki University Hospital, Helsinki, Finland; ^7^ Department of Diabetes, Central Clinical School, Monash University, Melbourne, VIC, Australia; ^8^ Neurology, University of Helsinki and Helsinki University Hospital, Helsinki, Finland; ^9^ Joslin Diabetes Center, Harvard Medical School, Boston, MA, United States; ^10^ Minerva Institute for Medical Research, Helsinki, Finland

**Keywords:** cerebral microbleeds, cerebrovascular disease, diabetic neuropathy, diabetic retinopathy, magnetic resonance imaging, neuroradiology, optic chiasm, type 1 diabetes

## Abstract

**Introduction:**

Diabetic neuropathy and diabetic eye disease are well known complications of type 1 diabetes. We hypothesized that chronic hyperglycemia also damages the optic tract, which can be measured using routine magnetic resonance imaging. Our aim was to compare morphological differences in the optic tract between individuals with type 1 diabetes and healthy control subjects. Associations between optic tract atrophy and metabolic measures, cerebrovascular and microvascular diabetic complications were further studied among individuals with type 1 diabetes.

**Methods:**

We included 188 subjects with type 1 diabetes and 30 healthy controls, all recruited as part of the Finnish Diabetic Nephropathy Study. All participants underwent a clinical examination, biochemical work-up, and brain magnetic resonance imaging (MRI). Two different raters manually measured the optic tract.

**Results:**

The coronal area of the optic chiasm was smaller among those with type 1 diabetes compared to non-diabetic controls (median area 24.7 [21.0-28.5] vs 30.0 [26.7-33.3] mm^2^, p<0.001). In participants with type 1 diabetes, a smaller chiasmatic area was associated with duration of diabetes, glycated hemoglobin, and body mass index. Diabetic eye disease, kidney disease, neuropathy and the presence of cerebral microbleeds (CMBs) in brain MRI were associated with smaller chiasmatic size (p<0.05 for all).

**Conclusion:**

Individuals with type 1 diabetes had smaller optic chiasms than healthy controls, suggesting that diabetic neurodegenerative changes extend to the optic nerve tract. This hypothesis was further supported by the association of smaller chiasm with chronic hyperglycemia, duration of diabetes, diabetic microvascular complications, as well as and CMBs in individuals with type 1 diabetes.

## Introduction

Diabetic neuropathy and kidney disease are well known neurovascular complications to type 1 diabetes, for which chronically high blood glucose is a major risk factor (1–4). During the first two decades of disease, nearly all people with type 1 diabetes show signs of diabetic eye disease (2), and half develop peripheral neuropathy (4). Further, diabetic kidney disease is a strong risk factor for other vascular complications in these individuals (5). However, other changes of the visual pathway in the central nervous system (CNS), consisting of the prechiasmatic optic nerve, optic chiasm, postchiasmatic optic tract, lateral geniculate body, visual cortex, and visual association cortex, are less well studied in this patient group. Some observations on functional visual pathway abnormalities have been described, showing associations between metabolic alterations, production of advanced glycation end products, oxidative stress, and hemodynamic changes (6). Interestingly, diagnostic tests such as visual evoked potential and optical coherence tomography angiography have detected functional and structural changes in the visual pathway among those with type 1 diabetes (7, 8). These changes may reflect early and irreversible loss of retinal ganglion cell axons *via* Wallerian degeneration (9) and raises the question of whether this hypothetical early atrophy of the proximal optic nerve could be detected by magnetic resonance imaging (MRI).

Nerve fibers from the nasal retina cross contralaterally in the optic chiasm. This structure enables vision from one side of both eyes to be rendered and interpreted by the occipital cortex of the opposite side (10). The chiasm can be delineated on coronal T1 reconstruction images with high precision, using standard imaging workstation tools (11). The most common presenting symptoms associated with chiasmatic syndrome are low visual acuity and bitemporal hemianopsia, often related to compression of the optic chiasm by pituitary and other parasellar masses (12, 13). A diminished optic chiasm is, however, a common finding in a range of disorders, such as multiple sclerosis, sarcoidosis, hydrocephalus, and compressive pituitary adenomas (10, 14, 15).

One neuropathological study (16) from 1965 observed 16 juvenile people with type 1 diabetes dying of diabetic neuroangiopathy after many years of diabetes is presented. In this post-mortem study, 5 showed a normal optic chiasm, 8 mild and 3 severe atrophy of the chiasm. Histological examination showed astrocyte proliferation as well as loss and degeneration of both the myelin sheaths and the axis cylinders of varying degrees. Some of the chiasms were almost completely destroyed and transformed into a cicatrix of glial and connective tissue. We found no other previous data of the size of optic chiasm in individuals type 1 diabetes.

We hypothesized that middle-aged individuals with type 1 diabetes without severe diabetic complications would have smaller optic chiasm than controls. We further hypothesized that the size of optic chiasm would correlate with non-CNS diabetic complications and signs of microvascular brain lesions.

Thus, our first aim was to compare the size of the optic chiasm on MRI in individuals with type 1 diabetes and healthy controls. Second, we studied whether diabetes-related covariates, including glycemic control, blood pressure and presence of diabetic microvascular complications would be associated with chiasmatic size. Third, we assessed whether chiasmatic size correlated with signs of cerebral small-vessel disease, including cerebral microbleeds (CMBs), lacunar infarcts, and white-matter hyperintensities (WMHs).

## Materials and methods

This study is a part of the prospective nationwide Finnish Diabetic Nephropathy (FinnDiane) Study. The design of the study has been described elsewhere in more detail (17). For this substudy, 191 subjects with type 1 diabetes were originally examined between 2011 and 2017. However, one subject with previous neurosurgery and two participants with multiple sclerosis were excluded from the analysis, resulting in a total of 188 subjects. A total of 30 heathy age and sex-matched controls were enrolled. The inclusion criteria for type 1 diabetes in this study was based on disease onset before the age of 40 and initiation of insulin treatment within one year of diagnosis. Exclusion criteria were any neurological symptom or disease, kidney replacement therapy or known coronary artery disease. None of the participants had a history or clinical manifestations of cerebrovascular disease. The study was carried out in accordance with the Declaration of Helsinki and the protocol was approved by the Ethics Committee of the Helsinki and Uusimaa Hospital District. Each participant gave written informed consent prior to participation.

All participants underwent a clinical study visit and comprehensive laboratory work-up at the FinnDiane research unit at Biomedicum Helsinki, including review of previous medical history, structured questions on lifestyle, body mass index (BMI) and blood pressure measurements. Blood samples were drawn to analyze concentrations of plasma creatinine, lipids and lipoproteins (total cholesterol, low-density lipoprotein [LDL], high-density lipoprotein [HDL] and triglycerides), high-sensitivity C-reactive protein (hs-CRP) and blood glycated hemoglobin (HbA_1c_) using standardized methodology. Diabetic kidney disease was defined as increased albumin excretion rate (≥200 μg min−1 or ≥300 mg 24 h−1) in two out of three urine collections. The CKD-EPI-formula was used to estimate glomerular filtration rate (eGFR) (18). The Early Treatment of Diabetic Retinopathy (ETDRS) scale was used to classify diabetic eye disease (19). Acquisition, evaluation and grading of retinal images have been described elsewhere in detail (20). Presence no to mild diabetic retinopathy was defined as ETDRS score ≤35, moderate to severe NPDR as ETDRS score 43–57 and PDR as ETDRS score ≥61 (19). Similarly, the Michigan Neuropathy Screening Instrument (MNSI) was used to classify diabetic neuropathy, with a score of ≥4 referring to manifest peripheral neuropathy (21).

Within one year of the work-up, all participants underwent a 3T brain MRI (Achieva, Philips, Best, The Netherlands) at the Medical Imaging Center at the Helsinki University Hospital. MR-sequences used were T1, T2, FLAIR, T1 MPRAGE, DWI, SWI, T2* and MRA TOF.

Regarding small-vessel disease, number of CMBs, white matter hyperintensities (Fazekas scale, with category ≥1 considered a significant burden) and lacunar infarcts were scored according to standardized criteria (22). This analysis was performed by a senior neuro-radiologist (J.M.) with >10 years of experience, who was blinded to clinical parameters and results of volumetric analysis.

**Figure 1:**
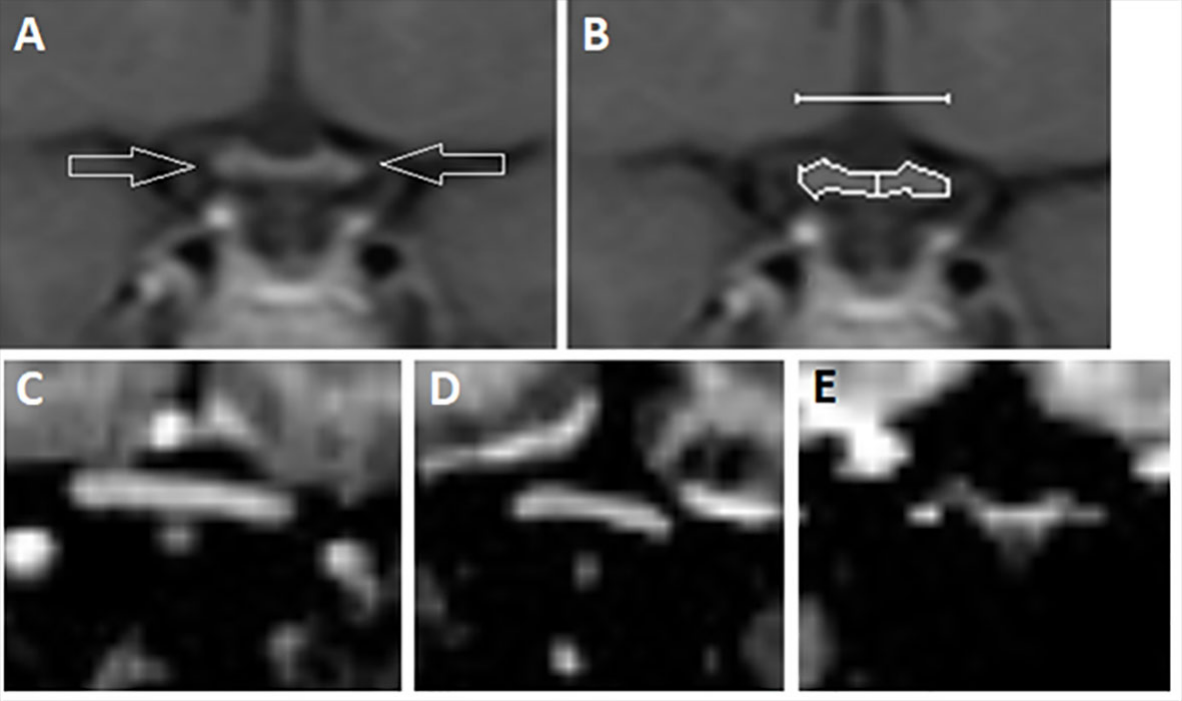
**(A)** Coronal T1 images of optic chiasm (arrows) were obtained with **(B)** width (upper line), height (vertical midline) and area (surrounding line) of the chiasm measured; **(C)** Example of chiasm of a healthy control; **(D)** Chiasm in a subject with type 1 diabetes and no microbleeds; **(E)** Chiasm in a subject with type 1 diabetes with both multiple cerebral microbleeds and received laser photocoagulation.

The optic pathway was interpreted and measured using reformatted coronal T1 MPRAGE images. Prechiasmatic optic nerves and postchiasmatic optic tracts were measured by their height (vertical diameter in coronal magnetic resonance images), and the area of the chiasm was measured by outlining its contours using the standard tracing tool of the Agfa Impax workstation (**Figure 1**). The chiasmatic area was measured a total of three times by each rater and the mean value of these was used, with results given as mm^2^. The height and width of the chiasm were also measured (mm). Two radiologists (J.M. and A.T.) performed the measurements independently blinded to clinical data.

### Statistical methods

All measured variables were tested for normality of distribution using Shapiro-Wilks’s test. Group comparisons of normally distributed variables were analyzed with two-sided Students t-test. Variables with non-normal distributions were tested with Mann-Whitney U test. Data are presented as mean ± SD (normally distributed) or median with interquartile range (non-normally distributed).

The achieved power of the group comparisons was calculated using G*Power software (version 3.1) with the following input parameters: *Post hoc* type of power analysis, two-tailed Mann-Whitney test, an effect size of 0.83, an alpha error probability of 0.05, and a sample size of 188 for the patient group and 30 for the control group. The achieved power was found to be 0.974.

For intra-rater reliability of the chiasmatic size measurements between the two raters, correlations between manual measurements were examined with Pearson’s correlation test when normally distributed and with Spearman’s test when non-normally distributed. Intraclass Correlation Coefficient (ICC) values were calculated.

We divided participants with diabetes into two groups according to previously observed CMBs: none (n=143) or at least one (n=45). Size of chiasm was compared between these two groups using Mann-Whitney U test. Similar comparison was performed between those with or without WMHs, lacunar infarcts, diabetic eye disease, received laser photocoagulation, diabetic kidney disease, and peripheral neuropathy.

MNSI scores for neuropathy were obtained in a subset for 126 of 188 subjects. In order to evaluate differences between these 126 participants and the remaining 62 participants in the type 1 diabetes group, these two subsets were tested for differences in age, sex, duration of diabetes, HbA_1c_, LDL-cholesterol, HDL-cholesterol, hs-CRP, microbleeds, WMHs, lacunar infarcts, diabetic eye disease and diabetic kidney disease using Mann-Whitney U test.

Furthermore, correlations between coronal chiasmatic area and clinical and laboratory parameters were analyzed in participants with diabetes using Spearman’s correlation test and linear regression analysis, where values of standardized coefficients were calculated in order to evaluate how many standard deviations chiasmatic area will change per standard deviation increase in a specific clinical parameter. These clinical parameters included duration of diabetes, age, HbA_1c_, LDL-cholesterol, HDL-cholesterol, hs-CRP, systolic and diastolic blood pressure. To analyze non-continuous parameters—including the presence of neuropathy (MSNI score ≥4), moderate or more severe retinopathy (ETDRS score ≥43), previously received laser photocoagulation or the presence of CMBs—we performed binary logistic regression analyses adjusting for age, duration of diabetes, HbA_1c,_ and BMI.

Power analyses were performed using G*Power software (version 3.1). Statistical analyses were used R (version 4.0.2). A two-sided p<0.05 was set as the threshold for statistical significance.

## Results

### Participants and descriptive data

Clinical characteristics and MRI findings of the 188 participants with type 1 diabetes and the 30 age- and sex-matched control subjects appear in **Table 1**. A total of 188 subjects with type 1 diabetes took part in the study (median age 40.0 years, interquartile range 33.0-45.1; 53.0% female). Median age of healthy controls was 38.4 (31.4-43.2) years with 56.5% of these being female (p for difference in age 0.433 and for sex 0.699). Median diabetes duration was 21.7 (18.3-30.9) years and median HbA_1c_ 66 ± 12 mmol/mol. Median systolic blood pressure was 130 (118-138) mmHg, cholesterol 4.4 (4.0-4.9) mmol/l, LDL 2.4 (2.0-2.9) mmol/l, HDL 1.50 (1.25-1.80) mmol/l and hs-CRP 1.16 (0.52-2.89) mg/l. Median 24-hour urinary albumin excretion rate was 13.4 (10.2-18.4) mg.

**Table 1:** Participant characteristics.

	People with type 1 diabetes (n=188)	Control subjects (n=30)	p for difference
Female sex, n (%)	100 (53)	17 (56.5)	0.699
Age, years	40.0 (33.0–45.1)	38.4 (31.4-43.2)	0.433
Diabetes duration, years	21.7 (18.3–30.9)	–	–
BMI, kg/m^2^	26.7 ± 4.2	24.5 ± 3.2	0.002
SBP, mmHg	130 ± 14	121 ± 11	0.001
DBP, mmHg	77 (71-82)	76 (74-85)	0.387
HbA_1c_, % (mmol/mol)	8.2 ± 1.1 (66 ± 12)	5.1 ± 0.2 (33 ± 2)	<0.001
Creatinine, µmol/L	68 (61-79)	74 (68-81)	0.067
Total cholesterol, mmol/L	4.4 (4.0-4.9)	4.6 (4.2-5.4)	0.178
LDL cholesterol, mmol/L	2.4 (2.0-2.9)	2.6 (2.3-3.3)	0.362
HDL cholesterol, mmol/L	1.50 (1.25-1.80)	1.46 (1.26-1.68)	0.398
Triglycerides, mmol/L	0.90 (0.68-1.38)	0.84 (0.69-1.26)	<0.001
Statin therapy, n (%)	42 (22)	0	0.002
Aspirin therapy, n (%)	15 (7.9)	0	0.232
Albuminuria, n (%)	30 (16)	0	0.018
Retinal photocoagulation, n (%)	44 (23)	0	0.002
Diabetic retinopathy, n (%)	155 (83)	0	<0.001
Current smoking, n (%)	15 (7.9)	5 (17)	0.118

Clinical characteristics and magnetic resonance imaging findings in participants with type 1 diabetes and control subjects matched for age and sex. BMI, Body-mass Index; SBP, systolic blood pressure; DBP, diastolic blood pressure; LDL, Low-density lipoprotein; HDL, High-density lipoprotein.

We were able to grade 187 fundal images from our 188 subjects. Diabetic retinopathy was present in 155 (83%) of the graded participants with type 1 diabetes. No to mild diabetic retinopathy (ETDRS score ≤35) was observed in 135 (72%) participants, moderate to severe NPDR (ETDRS score 43–57) in 23 (12%), and PDR (ETDRS score ≥61) in 29 (16%) participants. All 29 participants with PDR, 11 (48%) with moderate to very severe NPDR, and 4 (4%) with very mild or mild diabetic retinopathy had received laser photocoagulation, altogether 44 participants. There was no statistically significant difference in any tested parameters between the subset we were able to obtain MNSI scores from and the other participants in the type 1 diabetes group (p>0.05 for all). Median MNSI score was 1 (0-2).

### Chiasmatic measurements

None of the manually measured variables, including prechiasmatic optic nerves, coronal chiasmatic area, and postchiasmatic optic tracts, were normally distributed (p<0.05 for Shapiro-Wilks tests). Manually measured chiasmatic areas had a Pearson’s correlation coefficient of 0.86 (95% CI 0.82-0.89, p<0.001) between the two raters with an ICC of 0.822. Manually measured width had a Pearson’s correlation coefficient of 0.85 (95% CI 0.82-0.89). Between different raters, Pearson’s correlation coefficients varied between 0.43-0.65 in the pre- and postchiasmatic measurements.

**Figure 2:**
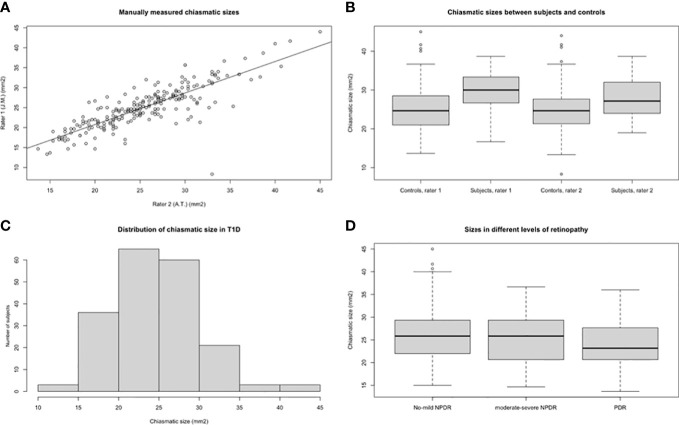
**(A)** Correlation plot comparing chiasmatic area measurements between two manual raters. **(B)** Boxplot of chiasmatic sizes between subjects and controls, measured by two independent raters. Rater 1 = J.M., rater 2 = A.T. **(C)** Distribution of chiasmatic sizes among individuals with type 1 diabetes. **(D)** Chiasmatic sizes in different levels of retinopathy.

Coronal chiasmatic area was smaller in participants with diabetes compared to healthy control subjects (median area 24.7 [21.0-28.5] vs 30.0 [26.7-33.3] mm^2^, p<0.001) (**Figure 2**). Median width was also smaller among those with type 1 diabetes, but this difference was not significant (median width 12.5 [11.7-13.3] vs 12.9 [12.1-14.1] mm, p=0.18). There was no measurable difference in median height of chiasma or median height of prechiasmatic nerves or postchiasmatic tracts between individuals with type 1 diabetes and controls, in any of these categories (1.7 vs 1.7 mm, p >0.05).

### Clinical and neuroimaging characteristics associated with chiasmatic size in individuals with type 1 diabetes

**Figure 3:**
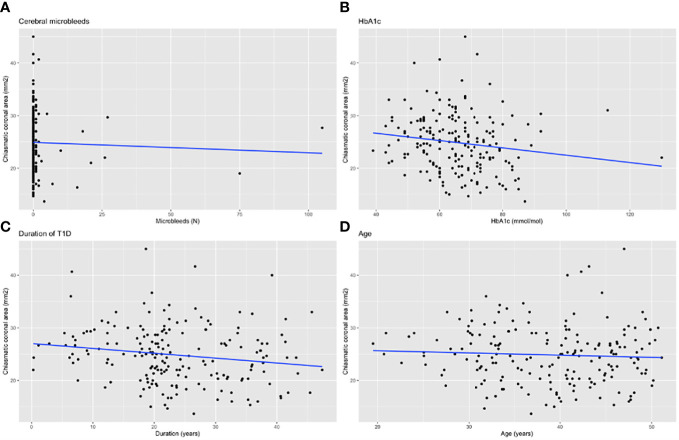
Correlation plots of **(A)** CMBs, **(B)** HbA1c, **(C)** duration of diabetes and **(D)** age with chiasmatic size. Estimated lines of linear regression analysis are plotted.

Duration of diabetes, BMI and HbA_1c_ were inversely associated with chiasmatic area. We observed no correlations between chiasmatic area and age, LDL-cholesterol, HDL-cholesterol, hs-CRP, systolic blood pressure, or diastolic blood pressure (**Table 2**, **Figure 3**).

**Table 2:** Linear regression parameters regarding cross-sectional area of optic chiasm in MR-images.

	B	Std. error	Beta	t value	p
**Age, years**	-0.0412	0.0540	-0.0741	-0.776	0.438
**Diabetes duration, years**	-0.924	0.0386	-0.211	-2.397	0.0175*
**HbA_1c,_ mmol/mol**	-0.0696	0.0317	-0.277	-2.20	0.029*
**BMI, kg/m^2^**	-0.202	0.0933	-0.153	-2.18	0.0315*
**Systolic BP, mmHg**	-0.0270	0.0272	-0.149	-0.994	0.322
**Number of microbleeds**	-0.00303	0.0410	-0.050	-0.738	0.461
**Number of WMHs**	-1.30	0.407	-0.0941	-1.399	0.163
**Number of lacunar infarcts**	-0.896	2.839	-0.0213	-0.316	0.753
**hs-CRP, mg/L**	-0.186	0.126	-0.106	-1.542	0.124
**Total cholesterol, mmol/L**	-0.0242	0.474	-0.00345	-0.051	0.959
**LDL-cholesterol, mmol/L**	1.64	0.985	0.120	1.66	0.099
**HDL-cholesterol, mmol/L**	-0.2078	0.521	-0.0291	-0.399	0.45
**eGFR, ml/min/1.73m^2^**	0.0100	0.00243	0.0278	0.412	0.618
**Triglyserides, mmol/L**	-0.733	0.543	-0.0976	-1.348	0.179

B and Beta representing unstandardized and standardized coefficients, respectively.

BMI, Body Mass Index; BP, Blood Pressure; WMHs, White Matter Hyperintensities; hs-CRP, high sensitivity C-reactive protein; LDL, Low Density Lipoprotein; HDL, High Density Lipoprotein; eGFR, estimated Glomerular Filtration Rate.

Compared to those without diabetic kidney disease, the chiasmatic area was smaller in patients with diabetic kidney disease (median 22.2 [19.2-26.7] vs 25.7 [22.0-29.5] mm^2^, p<0.001). The chiasmatic area was also smaller among individuals with ETDRS-score ≥43 versus ETDRS-score 43 (median 23.2 [20.3-26.4] vs 25.3 [21.8-29.0] mm2, p=0.040) as well as in individuals with peripheral neuropathy vs. those without (median 25.0 [20.6-28.8] vs 25.0 [17.8-26.49], p=0.041). Chiasmatic area was smaller among those subjects who had received laser photocoagulation (median 21.67 [18.3-25.08] vs 25.33 [22.00-29.00] mm^2^, p<0.001).

Median chiasmatic area was smaller among individuals with CMBs (23.5 [20.0-27.3] vs 25.3 [22.0-28.2] mm^2^, p=0.022) compared to those without. Subjects with WMHs had a median area of 23.7 [20.5-28.4] mm^2^ and without WMHs 25.7 [22.0-29.2] mm^2^ (p=0.17). No differences in chiasmatic size were observed when grouping by the presence of lacunar infarcts (median 25.2 vs 25.3 mm^2^, p=0.83).

In the binary logistic regression analysis, chiasmatic size was independently associated with the presence of peripheral neuropathy adjusting for age, HbA_1c_, diabetes duration and BMI (OR 0.83 per decrease of 1 mm^2^, 95% CI 0.69-0.98, p=0.032). In a similar model, we found an association between chiasmatic size and laser photocoagulation (OR 0.98 per decrease of 1 mm^2^, 95% CI0.975-0.995, p=0.004). There was no significant association between chiasmatic size and at least moderate diabetic eye disease (OR 0.95 per decrease of 1 mm^2^, 95% CI 0.89-1.01, p=0.096) or CMBs (OR 0.99 per decrease of 1 mm^2^, 95% CI 0.92-1.05, p=0.650) when age, HbA_1c_, diabetes duration and BMI were adjusted for.

## Discussion

The main finding of our study was that chiasmatic cross-sectional coronal area was smaller in subjects with type 1 diabetes than in healthy controls. Glycemic load measured as glycated hemoglobin, duration of diabetes and diabetic microvascular complications, including diabetic kidney disease, eye disease and peripheral neuropathy, were all associated with a smaller optic chiasm in people with type 1 diabetes. Moreover, the number of CMBs in individuals with type 1 diabetes correlated inversely with the size of the optic chiasm; those with at least one CMB were more likely to have a smaller chiasm. Our finding of the strong correlation of chiasmatic size with HbA_1c_ level and duration of type 1 diabetes, indicate a role of high blood glucose as a possible neurodegenerative agent also in the proximal visual pathway. We have earlier shown in individuals with type 1 diabetes, that long-term glycemic control is dependent on the brain tissue type, in accordance with these findings (23, 24). Based on their strong correlation with glycemic control, it was logical that we also found associations with chiasmatic size with neuropathy, diabetic kidney disease, and diabetic eye disease, because all these complications of diabetes are known to be related to glycemic control.

In this 1965 neuropathological study (16), severe atrophy of the optic chiasm was more common among the blind and visually impaired individuals with type 1 diabetes, while those with milder atrophy all had visual acuity of >0.5 or only a vision problem in one eye. Therefore, one very likely underlying cause of a small chiasm in diabetic patients is damage to nerves in the retina secondary to diabetic retinopathy or perhaps laser photocoagulation, thereby leading to atrophy of the optic nerves and the optic chiasm. Difference in chiasmatic size did appear when compared those with ETDRS<43 to those with more severe retinopathy, but this difference was even clearer after we observed those who had received laser photocoagulation. This later association was obvious even after HbA1c, duration, and BMI were adjusted. We observed similarly in this study (20), that CMBs associated with severe or proliferative form of diabetic eye disease, but not with less-than-severe forms of retinopathy.

Therefore, unsurprisingly, number of CMBs was associated with decreasing chiasmatic size among those with type 1 diabetes. In contrast, however, we found no association between other signs of microvascular parenchymal brain damage (lacunar infarcts and T2 hyperintensities) and size of the chiasm. Some potential explanations for our finding can be hypothesized, the most obvious being the connection between poor glycemic control and radical formation and their effect on developing peripheral neuropathy (25). This mechanism may be present also in the CNS, resulting in early neurodegeneration and thus reflecting correlation with other diabetes-related parameters, including CMBs. However, CMBs can also be a marker of early vascular neurodegeneration, partially causing the atrophy of optic chiasm and hypothetically also other parts of the CNS (26). We did not evaluate any other regions of the brain in our study, so it remains unclear if visual pathways are preferentially affected or whether the chiasmal atrophy is part of a more widespread atrophic process in the brain.

It remains also unclear whether smaller optic chiasm itself has any clinically observable consequences. Known symptoms related to a small chiasm are bitemporal hemianopsia and low visual acuity, but these are commonly related to compressive disorders, such as pituitary adenomas. Nevertheless, visual pathway abnormalities have been described in individuals with type 1 diabetes without diabetic eye disease with proximal optic nerve atrophy being one of the possible explanations (24). Future studies are needed to investigate whether a strict blood glucose control would prevent visual pathway abnormalities.

Certain technical aspects of chiasmatic measurements deserve consideration. In one study, chiasmatic areas were measured using a region of interest-protocol and coronal T1 images, achieving similar results in a healthy population, with a marginally smaller chiasmatic area in women than in men, and a decrease in the area among subjects over the age of 60 (11). In our study, the oldest subject was in his early fifties, and there was neither difference in chiasmatic sizes between subjects over the age of 50 and under the age of 30, nor any correlation between chiasmatic size and age as a continuous variable. Another study measured width and height but not coronal chiasmatic area in 77 healthy subjects, 20 subjects with visual impairment, and 13 subjects with visual impairment and funduscopic evidence of optic atrophy (27). In that study, the mean dimension of the optic chiasm was smaller in the visual impairment group, and an association between funduscopic optic atrophy and smaller chiasm was observed. The latest methodological study measured width and height, but not area, of chiasma (28). Unlike in the other studies, in that study the chiasm was measured using T2-weighted MRI sequences. In that study, the mean chiasmatic width was 13.63 mm ± 1.21 mm, a result very similar to ours.

Manual measurements between different raters showed good agreement in measuring the optic chiasm in our study, indicating that this method is reliable and repeatable. In agreement with earlier results (11), we found that the chiasmatic coronal area to be the most reliable measurement. In our study, prechiasmatic optic nerves, chiasmatic height and postchiasmatic optic tracts were not reliably measurable repeatedly. This is most likely due to the large voxel size in comparison to the relatively small target of interest, and these vertical measurements gained only certain quantitative values (3.3 mm, 2.5 mm, 1.7 mm or 0.9 mm). Furthermore, we found no difference in any of our pre- and postchiasmatic measurements or of chiasmatic height between those with type 1 diabetes and controls.

Our study has strengths. The samples size is large for this study question. We had strong phenotypic data on our participants and used standardized imaging and clinical assessment. Two raters performed the chiasmatic measurements independently, with good correlation in the coronal chiasmatic area. The method chosen to retrospectively measure the chiasm from routine 3D T1 MR reconstruction images can be considered as a strength as well as a weakness. More accurate measurements could have been possible from orbital-focused MRI protocols. The sequences used in our study are a common part of routine protocols in most brain MRI. This also explains why we were not technically able to measure size of prechiasmatic optic nerves or postchiasmatic optic tracts reliably, since the voxels are relatively large in comparison to the area of interest. Furthermore, as an inexorable phenomenon, MRI techniques and equipment have continued to develop since the time of the study (2011-2017) and the ability to detect CMBs and other microvascular pathologies has improved over time (29). The lack of OCT-A measurements and functional ophthalmologic data such as visual acuity is also a shortcoming.

In conclusion, chiasmatic cross-sectional coronal areas, reliably measured from brain MRI, were smaller in subjects with type 1 diabetes compared to non-diabetic subjects. A larger glycemic load was closely related with a smaller optic chiasm in people with type 1 diabetes. We showed an association between the chiasmatic size and diabetic microvascular complications, including diabetic eye disease, kidney disease and neuropathy, in these individuals. The number of CMBs correlated with the size of the optic chiasm in individuals with type 1 diabetes. Associations between chiasmatic size and diabetic neuropathy, as well as between chiasmatic size and previous laser photocoagulation treatment, remained also after adjusted for relevant cofounders.

## Data availability statement

The raw data supporting the conclusions of this article will be made available by the authors, without undue reservation.

## Ethics statement

The study protocol was carried out in accordance with the Declaration of Helsinki and was approved by the Ethics Committee of Helsinki University Hospital (HUS), ID: HUS/2184/2017. The patients/participants provided their written informed consent to participate in this study.

## Author contributions

AT and JM contributed to the study concept and design. AT and T-BC performed data analysis and interpretation. The manuscript was written by AT, with assistance from T-BC, CF, JM, JP, and DG. All authors reviewed and contributed to manuscript revisions. P-HG is the guarantor of this work and, as such, had full access to all the data in the study and takes responsibility for the integrity of the data and the accuracy of the data analysis. This study or parts of it has not been presented elsewhere. All authors contributed to the article and approved the submitted version.
